# Spontaneous subdural hematoma of the thoracolumbar region with massive recurrent bleed

**DOI:** 10.4103/0019-5413.49383

**Published:** 2009

**Authors:** Rafael Cincu, Francisco de Asis Lorente, David Rivero, José Eiras, José Ramón Ara

**Affiliations:** Department of Neurosurgery, Miguel Servet University Hospital, Zaragoza, Spain; 1 Department of Neurology, Miguel Servet University Hospital, Zaragoza, Spain

**Keywords:** Polycythemia, spine, subdural hematoma

## Abstract

Spinal subdural hematoma is a rare disorder and can be caused by abnormalities of coagulation, blood dyscrasias, lumbar puncture, trauma, underlying neoplasm, and arteriovenous malformation. We discuss an unusual case of an elderly woman who presented with spontaneous spinal subdural hematoma and developed massive rebleeding on the third day following initial evacuation of hematoma. This case illustrates that a patient with routine normal coagulation profile and adequate hemostasis can still harbor platelet dysfunction (in present case due to polycythemia) and later on can manifest as rebleeding and neurological deterioration.

## INTRODUCTION

Spinal subdural hematoma can be caused by abnormalities of coagulation, blood dyscrasias, lumbar puncture or trauma, underlying neoplasm, and arteriovenous malformation.^1^–^5^ In the absence of these underlying conditions, the occurrence of spinal subdural hematoma is extremely rare.^1^ We discuss a case of an elderly woman who had spontaneous spinal subdural hematoma and developed massive rebleeding on the third day following initial evacuation of hematoma.

## CASE REPORT

**Figure 1:**
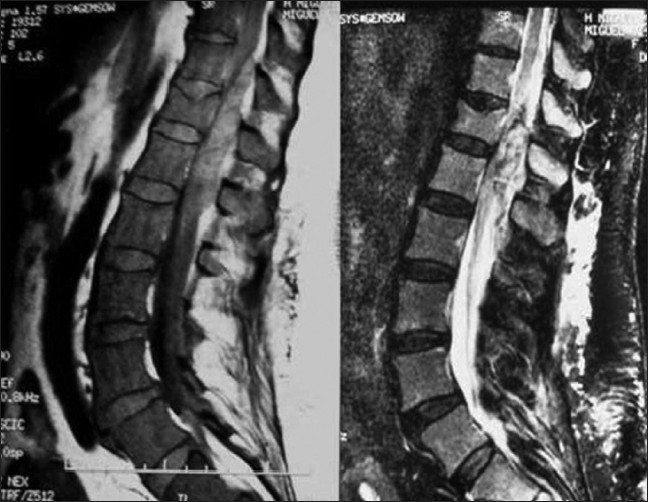
Sagittal MR image of thoracolumbar region showing relatively hyperintense lesion on T1 that becoming further hyperintense on T2 image with compression of the cord extending from D11 to L1 level

**Figure 2:**
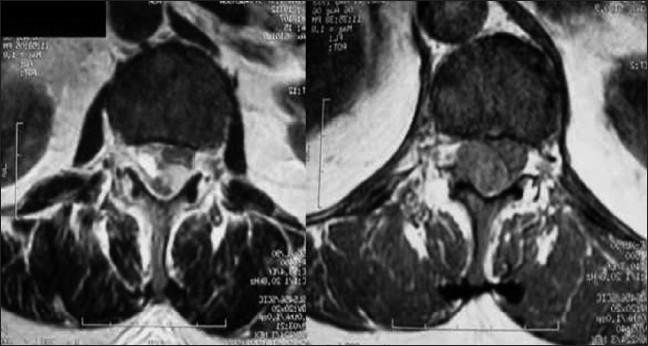
Axial MR image showing the biconvex nature of the lesion

**Figure 3:**
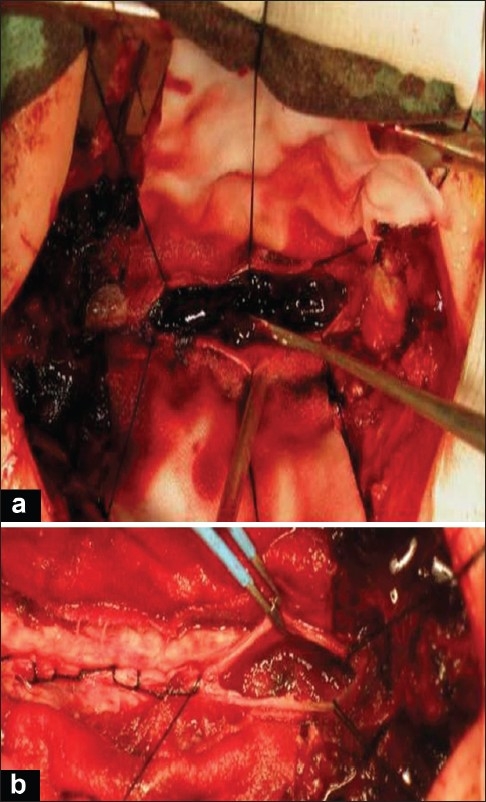
Intraoperative photographs, (a) showing intradural clotted blood, and (b) showing the thin clot adherent to the cord and hemostasis, note a piece of gel foam (gel foam was removed after achieving hemostasis).

**Figure 4:**
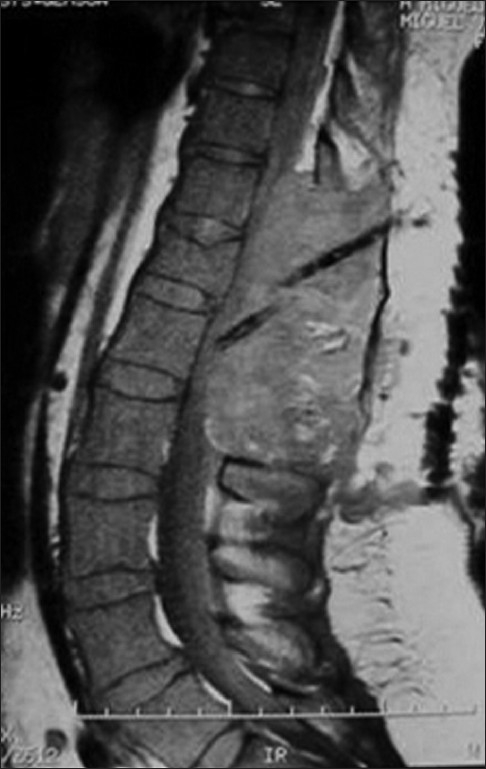
Postoperative saggital MRI of the thoracolumbar region (on the third day) showing massive collection of blood

A 73-year-old woman presented with a sudden onset of sensory loss below L1 level and weakness of left lower limb. There was no history of bowel or bladder disturbances and a history of trauma or anticoagulation therapy. Her general systemic examination was unremarkable. Neurologically she was conscious and oriented. Her cranial nerve examination was normal. Left lower limb was flaccid with grade 1/5 power and absent deep tendon reflexes. Right lower limb and both upper limbs were normal. Magnetic resonance imaging (MRI) of the spine showed evidence of extensive hemorrhagic collection extending from D11 to L1 level [Figures 1 and 2]. There was no evidence of vertebral fracture. A diagnosis of spontaneous spinal epidural hematoma was suspected. Routine blood investigations including routine coagulation profile were within normal limits and the hemoglobin was 15 gm%. The patient was planned for emergency D11 to L1 decompressive laminectomy and evacuation of hematoma. After opening the laminae, there was no blood; however, the dura was bluish and tense. The dura was opened, and there was thick clotted and altered blood [Figure 3a]. Hematoma was removed completely except a thin layer densely adhered to the cord. Complete hemostasis could be achieved after surgery though it took more time than usual [Figure 3b]. Drain was placed below muscular plain but without negative suction. By next day morning, she improved from grade 1/5 to grade 3/5 in left lower limb. She was doing well until next 36 hours after surgery when she deteriorated and became paraplegic within a span of 2 hours. Repeat MRI showed re-collection of hematoma extending from skin to the spinal cord [Figure 4]. She underwent urgent reexploration and evacuation of collection of hematoma. Detailed hematological evaluation showed evidence of polycythemia vera (mild splenomegaly that was confirmed using ultrasound), absolute erythrocytosis, platelet count-450×10^9^/L and prolonged bleeding and clotting time. The platelet transfusion was used to correct the platelet dysfunction. She received regular physiotherapy and, at 14 months follow–up, her power in lower limbs improved to 5/5 in right lower limb and 4/5 in left lower limb. She was able to carry activities of daily living independently and did not have any sphincter disturbances.

## DISCUSSION

The pathogenesis of spinal subdural hematoma (SSDH) is controversial and many hypotheses have been reported.^6^–^9^ According to one theory, the initial hemorrhage in the subarachnoid space is thought to be the primary lesion that subsequently ruptured into the subdural space, and the subarachnoid hemorrhage could be washed out by cerebrospinal flow,^6^–^9^ and this blood could be dissipated by the CSF faster in the subarachnoid space than in the subdural space.^9^ According to another theory, the spinal subdural hematoma may be related to a hemorrhage of the intracranial subdural space, and its expansion may be secondary to chronic changes.^6^,^10^ Rader proposed in 1955 that sudden and sharp increases in abdominal and thoracic pressures are transmitted intravascularly along the lateral spinal arteries and veins through the intervertebral foramens to the intraspinal segments of the vessels.^11^ Such a rapid increase in intravascular pressure cannot be neutralized by a simultaneous increase in spinal fluid pressure because of the shielding effect of the spinal column and ligaments, leading to the great disparity between the intravascular and extravascular pressures in the spinal canal and resulting in rupture of spinal vessels. This hypothesis was further supported by Minamide *et al*.^12^ In the present case, as most of these lesions are situated in lumbar region (up to 80%), they manifest as low back pain or cauda equine compression and associated motor and sensory deficits depending on the severity of compression.^5^,^13^–^15^ Usually there may be an associated history of minor trauma^16^ and sometimes with slow onset if the bleeding due to venous origin of the haematoma.^17^ Most of these lesions are located in a ventral location, but can occur posteriorly, laterally, and sometimes circumferentially.^18^ In our case, there was no history of trauma; however, there was associated coagulation anomaly. MRI is the investigation of choice and recognizes the blood products, a very important clue for the diagnosis of SSDH.^4^,^18^ MRI findings of high signal intensity lesion in both T1 and T2 sequences suggest the possibility of subdural hematoma although it can mimic the tumor-like cystic lesion of the cord.^4^ It has been described that epidural hematoma has a convex shape on sagittal and axial MRI, whereas subdural hematoma appears as concave on sagittal and irregular on axial MRI.^19^,^20^ However, it was difficult to identify the exact nature of the lesion in our case until surgery. SSDH is a surgical emergency, and requires surgical decompression as rapid surgical drainage of the subdural hematoma will be associated with the best prognosis especially in the cervical, thoracic, and thoracolumbar junctions of the spinal cord.^3^,^4^ It is difficult to evacuate the hematoma completely as often has extended and elongated configuration.^21^ Recently many reports have pointed out the possibility of spontaneous resolution of the SSDH^17^,^22^,^23^ and conservative treatment plays a role in the management of SSDH with stable neurological status.^4^,^24^ The neurological recovery depends on the duration of symptoms, severity of compression, and the aggressiveness of surgical intervention in symptomatic patients.^25^,^26^ In up to 54% cases, the defect was found in the hemostatic mechanism,^3^ and it is known that anticoagulation even when well controlled is not without risk.^13^ The patients with polycythemia vera can have both thrombotic and hemorrhagic complications.^27^,^28^

This case illustrates that patients with routine normal coagulation profile and adequate haemostasis can still harbor platelet dysfunction (in present case due to polycythemia) and can manifest as rebleed and neurological deterioration. This can be managed with urgent decompression and identification of the underlying pathology with adequate control of coagulation anomaly with good functional outcome.
